# Cryptococcal Protease(s) and the Activation of SARS-CoV-2 Spike (S) Protein

**DOI:** 10.3390/cells11030437

**Published:** 2022-01-27

**Authors:** Nozethu Mjokane, Maphori Maliehe, Olufemi S. Folorunso, Adepemi O. Ogundeji, Onele M. N. Gcilitshana, Jacobus Albertyn, Carolina H. Pohl, Olihile M. Sebolai

**Affiliations:** Department of Microbiology and Biochemistry, University of the Free State, 205 Nelson Mandela Drive, Park West, Bloemfontein 9301, South Africa; nmjokane@gmail.com (N.M.); maliehe.maphori@gmail.com (M.M.); foxxyphemmzy@gmail.com (O.S.F.); ogundejiao@ufs.ac.za (A.O.O.); gcilitshanaomn@ufs.ac.za (O.M.N.G.); albertynj@ufs.ac.za (J.A.); pohlch@ufs.ac.za (C.H.P.)

**Keywords:** cryptococcal infection, *Cryptococcus*, *Cryptococcus neoformans*, fluorogenic peptide, furin, protease, proteolytic cleavage, S1/S2 cleavage site, SARS-CoV-2, spike (S) protein

## Abstract

In this contribution, we report on the possibility that cryptococcal protease(s) could activate the SARS-CoV-2 spike (S) protein. The S protein is documented to have a unique four-amino-acid sequence (underlined, SPRRAR↓S) at the interface between the S1 and S2 sites, that serves as a cleavage site for the human protease, furin. We compared the biochemical efficiency of cryptococcal protease(s) and furin to mediate the proteolytic cleavage of the S1/S2 site in a fluorogenic peptide. We show that cryptococcal protease(s) processes this site in a manner comparable to the efficiency of furin (*p* > 0.581). We conclude the paper by discussing the impact of these findings in the context of a SARS-CoV-2 disease manifesting while there is an underlying cryptococcal infection.

## 1. Introduction

The novel coronavirus, severe acute respiratory syndrome coronavirus-2 (SARS-CoV-2, the etiological agent of the coronavirus disease of 2019, COVID-19), emerged in December of 2019 in Wuhan, China [1]. Upon colonisation, the virus manifests a pneumonia-like disease in which subjects have difficulties in gas exchange, making it hard to receive enough oxygen and expel carbon dioxide [2,3]. Patients may display mild symptoms and some may experience severe complications such as acute respiratory distress syndrome (ARDS) that may lead to multiple organ failure [4,5,6,7].

To achieve entry, the virus depends on the binding of the spike (S) protein to an angiotensin-converting enzyme (ACE2)-expressing cell [8]. The S protein has two functional subunits, i.e., the S1 and S2 subunits [1]. The proteolytic cleavage of the S protein is documented to create an unstable receptor-binding domain that, in turn, leads to membrane fusion and subsequent endocytosis [9]. Several host proteases such as the trypsin-like protease (e.g., TMPRSS2), endopeptidases, including members of the cathepsin family, have been documented to catalyse the activation of the S protein [10,11]. Of interest in the current study is the action of the protease furin, for which we synthesized a peptide mimetic of the S protein with a furin cleavage site. Jaimes et al. reported that the early characterisation of the S protein revealed a unique site, i.e., constituted by a sequence of four amino acids (underlined, SPRRAR↓S) at the interface between the S1 and S2 subunits, that serves as a potential cleavage site for furin [1,12,13,14,15].

From the above information, it is clear that activation can be achieved through the action of several proteases. This led us to theorise that microbial proteases could also activate the S protein [16], more so since proteases are characterised based on their catalytic mechanism that is defined by the nature of the nucleophilic amino acid in the active site [17,18,19].

The above is of particular interest, since there is documented evidence of the co-manifestation of SARS-CoV-2 and microbial infectious agents such as *Cryptococcus* (*C.*) *neoformans* [20,21,22]. To illustrate this, In Jiangsu, China, the early assessment of COVID-19 patients for microbial co-infections (i.e., within one to four days of SARS-CoV-2 infection) showed that over 90% of such patients were infected with other respiratory pathogens [16,23]. These case studies and associated literature reviews highlight the potential risks of pneumonia-causing microbes aggravating COVID-19. In particular, these microbes could secrete proteases that may also contribute to intensifying the sporadic viral activation. *C. neoformans* is known to purposefully secrete several proteases that skew the immune response towards tissue damage to promote its invasion [24,25,26,27,28,29,30,31,32]. This quality is also associated with *Aspergillus* (*A*.) *fumigatus*, another COVID-19-related mycotic agent [33]. In their paper, Kogan et al. reported that *A. fumigatus* could breach the alveolar epithelial cell barrier by secreting proteases that act in concert to disrupt the actin cytoskeleton and destroy cell attachment to the substrate by impairing focal adhesions [34].

One of the hydrolytic enzymes secreted by *C. neoformans* is an uncharacterised protease, which, similarly to furin, has serine as the nucleophilic amino acid in the active site [25]. Based on the latter, we sought to analyse the efficiency of cryptococcal protease(s) (secreted into the cultivation media) to cleave the mimetic peptide *viz*. MCA-ASYQTQTNSPRRARSVASQS-lys9(DNP) (SARS-CoV-2 S1/S2), which has a cleavage site for furin. The biochemical efficiency of cryptococcal protease(s) was then compared to that of commercially sourced furin.

## 2. Materials and Methods

### 2.1. Materials

Yeast extract, malt extract, peptone, glucose, microbiological agar, Hepes, Triton X-100, CaCl_2,_ and 2-mercaptoethanol were obtained from Merck (Johannesburg, GP, South Africa). Yeast nitrogen base (YNB) and Pierce^TM^ Colorimetric Protease Assay Kit were obtained from Thermo Fisher Scientific (Johannesburg, GP, South Africa); 50 mL centrifuge tubes and 2 mL plastic tubes were obtained from Lasec (Johannesburg, GP, South Africa), as well as a haemocytometer (Marienfeld, BW, Germany). Black, sterile disposable 96-well flat-bottom microtiter plates were obtained from Greiner Bio-One (Frickenhausen, BW, Germany). Recombinant furin was acquired from New England Biolabs (Ipswich, MA, USA). The mimetic peptide ASYQTQTNSPRRARSVASQS (corresponding to the amino acid sequence of SARS-CoV-2 S1/S2) containing the 7-methoxycoumarin-4-yl acetyl/2,4-dinitrophenyl (MCA/DNP) FRET pair was synthesised by Biomatik (Wilmington, DE, USA). VICTOR Nivo multimode microplate reader was purchased from PerkinElmer (Waltham, MA, USA).

### 2.2. Collection of Cryptococcal Protease(s) and Protease Assay

The standard cryptococcal reference strain, i.e., *C. neoformans* H99 (maintained as a culture at the University of the Free State, South Africa), was used in the study. This organism was grown on yeast–malt extract (YM) agar (3 g/L yeast extract, 3 g/L malt extract, 5 g/L peptone, 10 g/L glucose, 16 g/L agar). The plate was incubated for 24 h at 30 °C. After 24 h, fungal colonies were scooped with an inoculation loop and inoculated into a 50 mL centrifuge tube that contained 25 mL of fresh, sterile YNB (67 g/L) broth that was supplemented with glucose (4%; w/v). The flask was then incubated for 36 h at 30 °C while being agitated at 160 rpm on an orbital shaker. After 36 h, the cell density of the culture was determined using a haemocytometer. One mL of the culture media (containing cryptococcal cells) was dispensed into a 2 mL plastic tube. The tube was centrifuged at 1000× *g* (5 min at 30 °C) in order to pellet the cells and mobilise cryptococcal protease(s) into the supernatant.

To confirm mobilisation of cryptococcal protease(s) into the supernatant, the supernatant was tested with a Pierce^TM^ Colorimetric Protease Assay Kit in accordance with the manufacturer’s protocol. Three independent experiments were carried out.

### 2.3. Fluorogenic Assay: Proteolytic Cleavage of the SARS-CoV-2 Spike (S) Protein

Our protocol was modified from papers by Jaimes et al. [14,35]. The prepared reaction conditions were reported to activate the hydrolysis of most substrates by furin [36] and, by extension, other serine-based proteases. Moreover, the used substrate contained an amino acid sequence (underlined, SPRRAR↓S) that is highly susceptible to furin hydrolysis, as any mutation to the sequence would impair furin hydrolysis [14,35,36].

A reaction mixture for the synthesized fluorogenic mimetic peptide was carried out in a 100 μL buffer solution (pH 7.5) composed of (1) 100 mM Hepes, (2) 0.5% Triton X-100, (3) 1 mM CaCl_2_, and (4) 1 mM 2-mercaptoethanol. Furin was diluted to 10 U/mL, and 0.5 μL was added to the reaction mixture. In a separate experiment, 0.5 μL of the cryptococcal supernatant was added.

Reactions were performed at 30 °C, and a fluorometer measured fluorescence emission every minute for 45 min. Fluorescence intensity was tracked over this time interval using the wavelength settings, i.e., excitation (λ355 nm) and emission (λ405 nm). Six independent experiments were carried out, and the means Vmax was calculated.

### 2.4. Statistical Analyses

For each study, unless stated otherwise, three independent experiments were performed. No technical repeats were included for each independent experiment. The GraphPad Prism software, version 8.3.1 for windows was used to calculate mean values and the standard error of the means (SEM) (GraphPad Software, San Diego, CA, USA; www.graphpad.com) (accessed on 10 December 2021). Where appropriate, the same programme was used to perform the multiple comparison test using Tukey’s test as an option.

## 3. Results

### Cryptococcal Protease(s) Activate the Spike (S) Protein

**Figure 1:**
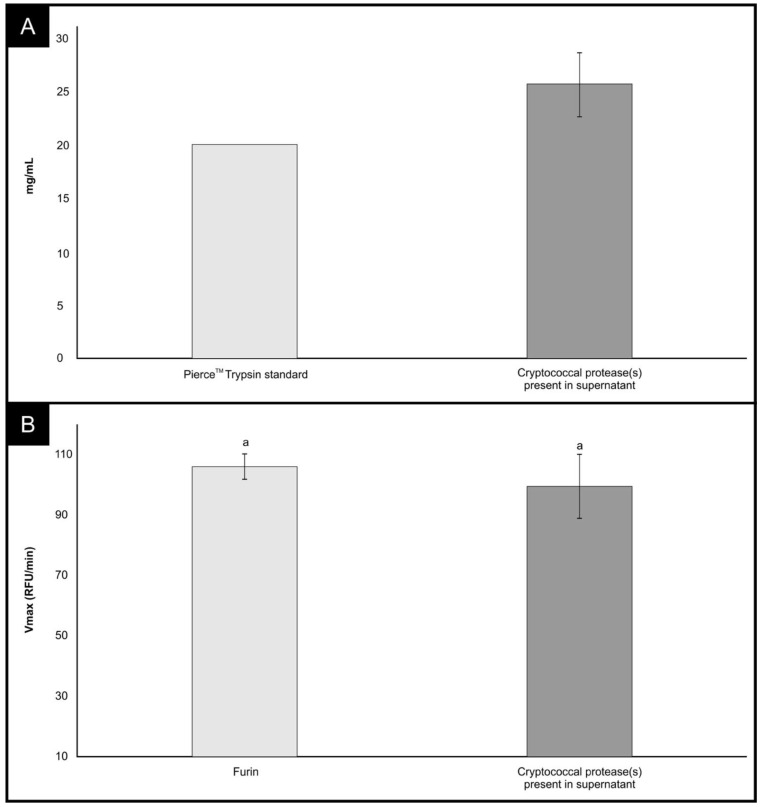
(**A**) The detection of cryptococcal protease(s) in the supernatant obtained from cryptococcal cultivation media, i.e., YNB broth. The depicted cryptococcal data were obtained from three independent experiments. (**B**) A measure of the proteolytic reaction following the cleavage of a fluorescent peptide viz. TNSPRRARSVA (SARS-CoV-2 S1/S2), by furin or cryptococcal protease(s) present in the supernatant. The depicted data were obtained from six independent experiments. Error bars represent SEM images. The subscript “a” indicates the data is not significantly different at *p* > 0.05.

Figure 1A summarizes the analysis results of the supernatant for the detection of cryptococcal proteases. The supernatant was collected from cells that had reached a cell density of 6.65 × 10^6^ cells/mL (SEM = 7.51 × 10^5^) after 36 h. It was estimated that approximately 25 mg/mL of protease(s) was present in the supernatant. This amount was extrapolated from the standard curve prepared using the trypsin standard provided in the Pierce^TM^ Colorimetric Protease Assay Kit.

**Figure 2:**
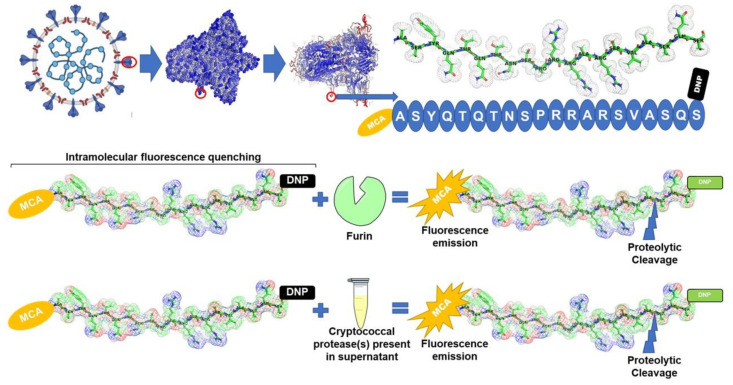
Based on the results depicted in Figure 1B, it seems the unique furin cleavage site (SPRRAR↓S) at the interface between the S1 and S2 sites may be efficiently cleaved by cryptococcal protease(s). The figure was built using BioRender.com.

More importantly, we show in Figure 1B that cryptococcal protease(s) targeted the unique four-amino-acid sequence (SPRRAR↓S) at the interface between the S1 and S2 sites. This unique site has been reported to be a potential cleavage site for furin, as mutations at this site impair furin-mediated processing [14,35,36]. We showed that cryptococcal protease(s) mediated the proteolytic cleavage of the fluorogenic peptide in a manner that was comparable to the efficiency of furin (*p* > 0.581). The biochemical peptide cleavage assay used in this study is well established in assessing the activation of the spike (S) protein. To this point, Jaimes et al. [14] reported that the assay was used to screen the influenza viruses [37] and feline coronavirus and MERS-CoV [38,39]. Taken together, the data presented herein suggest that other classes of proteases, including microbial proteases, could potentially activate the SARS-CoV-2 S1/S2 site (Figure 2). An important point to consider, as highlighted by Jaimes et al., is conceivable that a purified full-length SARS-CoV-2 S protein might not be cleaved in a similar manner (due to differences in conformation as it may not resemble the original folding of the full-length protein) to the memetic peptide used in the current study [14]. It is now prudent to isolate and identify these potential furin-like proteases from the *C. neoformans* secretome and to validate the work using a full-length S protein.

## 4. Discussion

SARS-CoV-2 infection begins with the inhalation of viral particles and the subsequent lodging in the alveolar space [40,41]. The respiratory route also serves as a portal of entry for other pneumonia-causing microbes, including *C. neoformans*. Therein, these microbes must successfully overcome the mammalian temperature barrier and scavenge available nutrients to support their growth. Therefore, it is not surprising that case reports are documenting the co-existence of SARS-CoV-2 with pneumonia-causing microbes [42,43]. In their study, Zhu et al. [23] reported that underlying microbial infections increase the severity of SARS-CoV-2 infection.

One of the attributes of invading pathogens causing infection in humans is the ability to degrade the epithelial tissue and penetrate the endothelial layers to reach the neighbouring tissues, acquire nutrients, disseminate, invade, and colonise peripheral tissues. Similar to every other infectious pathogen, *C. neoformans* secretes multiple peptidases and metalloproteinases that aid the cleavage of connecting tissue and epithelial matrixes, leading to tissue invasion and dissemination into the CNS and, as such, strains with higher protease secretion have been identified with more virulence [24,26,44,45]. The proteolytic degradation of defending proteins can potentiate allergic and immunoinflammatory responses such as cytokine release, kinin, and other plasma proteins induced by allergens, toxins, and pathogen-releasing peptidases [46]. This was evidenced in the work of Chen et al. [32] when extracellular proteases were detected in various isolates of *C. neoformans* with the ability to degrade human IgG and complement factor 5 in the microbial culture [32]. The most significant of this was the degradation of the plasma protease inhibitors—keepers of multi-organ proteases. Failure to keep the tissue proteases in check led to the sporadic release and activation of tissue proteases, further deteriorating the connective tissues and endothelial integrity.

Serine proteases are produced entirely in almost all the body tissues and transported in the plasma. The incessant activation of this protease due to compromising the plasma protease inhibitor contributes grossly to the activation of S protein of SARS-CoV-2 and aggravates the COVID-19 disease conditions. It is on this platform that we raised a concern. To corroborate this effect, we had earlier suggested that the transmembrane migration and “Trojan horse” feature of the infectious cryptococcal cell may be utilised by the extrapulmonary circulatory SARS-CoV-2 viral particles for transmedulla migration and colonisation [16].

As part of our effort to understand the co-morbidity of cryptococcosis and COVID-19, we unravelled the potential role of *C*. *neoformans* extracellular secretomes to activate the SARS-CoV-2 S protein. Hitherto, a serine protease, cathepsin L and B, and plasmin have been identified as endogenous proteases stimulating SARS-CoV-2 cellular endocytosis and are potential drug targeting sites [47]. Furthermore, the sequestration of neutrophils into the infected area promoted a preponderant level of granulocyte-derived serine proteases, which directly created an imbalance between the plasma protease inhibitor and the tissue proteases. On the other hand, the microbial proteases may have further contributed to this imbalance, increasing viral activation and tissue penetration, leading to hyper-inflammatory disorders, tissue atrophy/dystrophy, ARDS, and cerebral encephalitis.

The hyper-inflammatory response to cryptococcal antigens promoted epithelial damage, tight junction gap enlargement, and endothelial cell damage. In fact, *C. neoformans* can release proteases, ureases, and phospholipases to invade endothelial tissues, penetrate the BBB, and weaken tight intercellular junctions [48]. Consequently, plasma proteins are mobilised to the site of infection in response to the inflammation. One such example is the release of kinins to recruit granulocytes [49,50]. Apparently, the release of kinins induced by the fungi protease elevates vascular permeability that indirectly supplies nutrients to the pathogen and helps with fungi colonisation. In light of this, cryptococcal and SARS-CoV-2 co-infection may increase the antigen recognition and mobilise phagocyte engulfment of the fungi and pre-activated viral particles via the damaged endothelial barrier into the peripheral organs, including the CNS. The co-localisation and colonisation of the two pathogens in the medullar cortex may intensify the pathophysiological conditions associated with cryptococcal patients infected with COVID-19.

## 5. Conclusions

This pilot study detailed the first empirical data that were formative because we shed light onto the possible role of microbial proteases in activating the S protein. The latter allowed for an expansion of our understanding into COVID-19-associated mycosis. It is now prudent to conduct in vitro (using a virosome) and in vivo (using a laboratory animal) studies to assess if microbial proteases could also contribute to viral activation. Such a study should include a protease inhibitor and antifungal. This would assist in establishing sufficient merit to clear the underlying microbial infection in order to improve the vaccine’s response.

## Data Availability

The data presented in this study are available in the article.
